# Variability among the Most Rapidly Evolving Plastid Genomic Regions is Lineage-Specific: Implications of Pairwise Genome Comparisons in *Pyrus* (Rosaceae) and Other Angiosperms for Marker Choice

**DOI:** 10.1371/journal.pone.0112998

**Published:** 2014-11-18

**Authors:** Nadja Korotkova, Lars Nauheimer, Hasmik Ter-Voskanyan, Martin Allgaier, Thomas Borsch

**Affiliations:** 1 Institut für Biologie/Botanik, Systematische Botanik und Pflanzengeographie, Freie Universität Berlin, Berlin, Germany; 2 Dahlem Centre of Plant Sciences (DCPS), Berlin, Germany; 3 Botanischer Garten und Botanisches Museum Berlin-Dahlem, Berlin, Germany; 4 Institute of Botany, National Academy of Sciences of Republic Armenia, Yerevan, Armenia; 5 The Berlin Center for Genomics in Biodiversity Research (BeGenDiv), Berlin, Germany; The New York Botanical Garden, United States of America

## Abstract

Plastid genomes exhibit different levels of variability in their sequences, depending on the respective kinds of genomic regions. Genes are usually more conserved while noncoding introns and spacers evolve at a faster pace. While a set of about thirty maximum variable noncoding genomic regions has been suggested to provide universally promising phylogenetic markers throughout angiosperms, applications often require several regions to be sequenced for many individuals. Our project aims to illuminate evolutionary relationships and species-limits in the genus *Pyrus* (Rosaceae)—a typical case with very low genetic distances between taxa. In this study, we have sequenced the plastid genome of *Pyrus spinosa* and aligned it to the already available *P. pyrifolia* sequence. The overall *p*-distance of the two *Pyrus* genomes was 0.00145. The intergenic spacers between *ndhC–trnV*, *trnR–atpA*, *ndhF–rpl32*, *psbM–trnD*, and *trnQ–rps16* were the most variable regions, also comprising the highest total numbers of substitutions, indels and inversions (potentially informative characters). Our comparative analysis of further plastid genome pairs with similar low *p*-distances from *Oenothera* (representing another rosid), *Olea* (asterids) and *Cymbidium* (monocots) showed in each case a different ranking of genomic regions in terms of variability and potentially informative characters. Only two intergenic spacers (*ndhF–rpl32* and *trnK–rps16*) were consistently found among the 30 top-ranked regions. We have mapped the occurrence of substitutions and microstructural mutations in the four genome pairs. High AT content in specific sequence elements seems to foster frequent mutations. We conclude that the variability among the fastest evolving plastid genomic regions is lineage-specific and thus cannot be precisely predicted across angiosperms. The often lineage-specific occurrence of stem-loop elements in the sequences of introns and spacers also governs lineage-specific mutations. Sequencing whole plastid genomes to find markers for evolutionary analyses is therefore particularly useful when overall genetic distances are low.

## Introduction

Clarifying species limits and reconstructing phylogenetic relationships in clades with recently diverged species is challenging. Levels of genetic divergence are often low while at the same time large numbers of samples need to be analysed. The same applies to analysing phylogeographic patterns, where many individuals from different populations need to be included. Due to the often complex modes of speciation in angiosperms, evidence from uniparentally inherited organellar genomes and the recombined nuclear genome is needed to unravel evolutionary histories [1]–[3]. This is also the case in the genus *Pyrus* where — like in many Rosaceae — polyploidy, hybridization, and reticulate evolution occur. Estimates of *Pyrus* diversity vary between 50 and 80 species [4], [5] and 20 taxa alone have been described from the southern Caucasus [6], [7]. Similarly, the numbers of accepted species differ between treatments as a consequence of poorly understood species limits. *Pyrus* is a typical case for evolutionary and taxonomic analyses of diverse species groups in flowering plants that require the inclusion of hundreds of individuals. Before entering into large-scale sampling, we were interested to find the genomic regions with the best information potential for generating haplotype networks and inferring phylogenetic relationships. In this study, we focus on the plastid genome.

Along the same line of argumentation, Shaw et al. [8], [9] inspired to employ a broader spectrum of noncoding and rapidly evolving plastid markers in phylogenetic analyses of closely related species. Shaw et al. [8] sequenced a wide range of plastid markers for three species across angiosperms and later compared plastid genome pairs of three lineages of angiosperms (*Atropa* and *Nicotiana* for the asterids, *Lotus* and *Medicago* for the rosids, and *Oryza* and *Saccharum* for the monocots) [9]. Their studies resulted in a set of 32 regions that ranked highest in their number of potentially informative characters (defined as sum of substitutions, indels and inversions following [8] and abbreviated as “PICs”). This set was consequently suggested to generally contain the most variable and phylogenetically most informative genomic regions in angiosperm plastid genomes. However, the question remains how to best select four or five of the total top 32 regions, as many species-level evolutionary studies require.

Noncoding genomic regions such as introns and spacers often contain stem-loops and other specific structural elements that can be highly dynamic and are AT-rich. This results in a mosaic-like pattern of conserved and variable elements [10]. Considering that certain stem-loop elements within given introns and spacers are often unique to restricted lineages [11], [12], lineage specificity in the overall variability of genomic regions is to be expected. In several recent comparative analyses of angiosperm plastid genomes [13], [14] different genomic regions were depicted as the most variable. Nonetheless, these results need to be considered with care because some of the respective authors worked with pairs of hardly differentiated genomes while others had pairs of genomes with high *p*-distances. We expect that taxon-specific differences caused by certain sequence elements will be less prominent when more distant genomes are studied.

Next-generation sequencing techniques greatly facilitate the analysis of whole plastid genomes [15]–[17]. To date, phylogenomic studies of plastid genomes in land plants often just relied on concatenated sequences of the conserved genes, neglecting the information from the noncoding regions. In other cases, the authors included rather few taxa for which plastid genome sequences were automatically assembled from the respective 454 or Illumina runs, without completing parts of low coverage or areas with difficulties to obtain correct sequences. However, especially those might be informative at and below the species level (e.g., AT-rich stretches of DNA including microsatellites) [18]–[20]. On the other hand, there are recent studies which used completely annotated plastid genomes to detect infraspecific variability in species of *Olea* [21], *Colocasia* [22], or *Phalaenopsis* [23], or to find genomic regions with the highest number of potentially informative characters in more distant genome pairs of angiosperm genera [9], [24]–[26].

We have sequenced the plastid genome of *Pyrus spinosa* using 454 pyrosequencing in order to compare it with the published plastid genome sequence of *P. pyrifolia* [27]. In our *Pyrus* genome pair, the proportion of sites at which the two sequences are different (*p*-distances) is almost 10-fold lower than in the genome pairs studied by Shaw et al. [9]. For further comparison, we selected three fully annotated plastid genome pairs using the criterion of low *p*-distances (≤0.005) similar to *Pyrus*. Here we wanted to represent another rosid pair (*Oenothera parviflora* and *O. argillicola*; Onagraceae), an asterid pair (*Olea europaea* and *O. woodiana*; Oleaceae) and a monocot pair (*Cymbidium tortisepalum* and *C. sinense* (Orchidaceae).

The goals of this study were (1) to find the most variable regions of the *Pyrus* plastid genome and to propose plastid markers for species-level evolutionary studies in *Pyrus*, (2) to assess the variability of plastid genome regions based on comparable genome-pairs with overall low *p*-distances (0.0005 to 0.005) in major lineages of angiosperms, (3) to clarify if there are universal or lineage-specific rankings of variability within the group of about 35 top variable genomic regions, and (4) to evaluate if there are lineage specific differences in molecular evolutionary patterns that could cause the variability of genomic regions.

## Material and Methods

### DNA extraction, 454 pyrosequencing, genome assembly and annotation

*Pyrus spinosa* was sampled from the living collection of the Botanical Garden Berlin-Dahlem (Acc. No. 248458110, IPEN-Nr. TR-0-B-2484581, origin: Turkey: Kastamonu, Pontic Mountains around Küre, leg.: Ern, Krone 7145, 9/1981, voucher at B). The leaf tissue was silica-dried and total genomic DNA was extracted using the NucleoSpin Plant II kit (Macherey Nagel) according to the manufacturer's instructions.

Shotgun sequencing from total genomic DNA was performed on a Roche 454 GS-FLX Titanium sequencer (Roche Applied Science, Indianapolis, Indiana, USA). The 454 run (1/4 plate) resulted in 120,255 reads with an average of 400 bp after removing the adaptor sequences.

An initial mapping assembly with MIRA 4 [31] using *Pyrus pyrifolia* as reference resulted in 4191 reads mapped to a single contig with an average coverage of 13.44. However, reads with larger indels, not occurring in the reference, were not incorporated into the contigs what lead to an incorrect genome sequence. To remove the bias of the reference sequence, the reads were *de novo* assembled to contigs using the Roche GS *De Novo* Assembler (Newbler) v.2.6 which resulted in 836 large contigs (N50 = 829), and with Mira 4 [28], which resulted in 1125 large contigs (N50 = 1072, N90 = 538, N95 = 519). All these contigs were mapped on the *Pyrus pyrifolia* plastid genome (GenBank acc. no. NC015996; Terakami et al. [27]) using Geneious 7 to produce a consensus sequence. The combined method of mapping *de novo* contigs recovered nine indels (maximum length 71 bp), which were not found with mapping alone. Finally the second inverted repeat was manually inserted into the consensus sequence.

The positions of protein coding genes, rRNAs, tRNAs and the inverted repeats were annotated with the help of DOGMA [29] and Geneious 7. All coordinates of exons, reading frames and the positions of tRNAs were manually checked by aligning the respective genes of *Nicotiana tabacum* L. (NC001879) to the *Pyrus spinosa* sequence in PhyDe [30] because DOGMA tends to incorrectly place the start and stop codons and often does not annotate small exons. In case of more deviating gene sequences (e.g. *matK* or *ycf1*), the *Pyrus* gene sequences were translated to amino acid sequences to correctly annotate the reading frame.

#### Verification by Sanger sequencing

Pyrosequencing is limited in that the exact number of nucleotides within longer homonucleotide stretches (polyAs or polyTs) cannot be reliably determined [16], [31]. Our initial assembly contained several homonucleotide stretches and AT-rich sequence motifs. In our data, ambiguously called bases were frequent in homonucleotide stretches with more than six of the same nucleotides. To validate the sequence in such parts, we applied the Sanger method (electrophoresis was done at Macrogen Europe, Amsterdam, The Netherlands). Primers for amplification and sequencing were taken from the literature or designed in this study (see Table S1). Pherograms were checked by eye for peaks and corresponding quality scores to ensure that the polyA/T stretch was correctly read. All Sanger sequencing reads were unambiguous with no overlapping peaks after the polyA/T stretches. The respective reads were aligned with the previously assembled genome sequence in Geneious 7 and the consensus sequence was corrected accordingly. The *Pyrus spinosa* plastid genome sequence is available in EMBL under accession HG737342.

#### Pairwise genome comparisons and calculation of sequence divergence

In addition to *Pyrus*, we took three other plastid genome pairs from published sources to represent closely related species, a further rosid genus, an asterid and a monocot genus. Genome sequences had to be complete and fully annotated. The aligned genome pairs had to show an overall distance of *p*<0.005 (Table 1). All genome sequences were aligned in PhyDe using a motif alignment approach [32], [33]. The pairwise alignments are provided as File S1, S2, S3, and S4.

**Table 1:** GenBank accession numbers and references for the plastid genomes used in this study.

Species	GenBank accession number	Reference
*Pyrus spinosa*	HG737342	this study
*Pyrus pyrifolia*	NC015996	Terakami et al. [27]
*Cymbidium tortisepalum*	NC021431	Yang et al. [24]
*Cymbidium sinense*	NC021430	Yang et al. [24]
*Oenothera parviflora*	NC010362	Greiner et al. [66]
*Oenothera argillicola*	EU262887	Greiner et al. [67]
*Olea woodiana*	NC015608	Besnard et al. [68]
*Olea europaea*	NC015401	Besnard et al. [68]

Sequences of all introns and intergenic spacers larger than 100 bp were extracted from the alignments. The number of single nucleotide polymorphisms (SNPs) and indels for each sequence pair were counted with a script in R (v. 3.0.2). PICs were then determined in the sense of Shaw et al. [8] as the sum of all substitutions and indels. *P*-distances (proportion of differing nucleotide sites in the two sequences compared) of the regions were calculated by dividing the number of SNPs by the length of the regions without counting indel positions. The two parts of the *trnK* intron were analysed separately.

To assess the *p*-distances of the genome pairs used by Shaw et al. [8], we have aligned the genomes of *Lotus japonicus* (NC002694) and *Medicago truncatula* (AC093544); *Nicotiana tabacum* (NC001879) and *Atropa belladonna* (NC004561.1); *Saccharum* hybrid (NC005878) and *Oryza sativa* (NC008155) using MAFFT v. 7 [34], and calculated the *p*-distances of these genomes using PAUP* v. 4.0b10 [35].

To compare the whole genome variability apart from specific regions, a sliding window approach was performed counting the number of SNPs and indels and calculating the AT-content for 500 bp slots of the consensus sequences. The genome comparisons were visualized using Circos v. 0.64 [36].

### Molecular evolution within genomic regions

In order to assess the role of the base composition in variable sequence parts, i.e., indels and nucleotides around SNPs, we calculated their AT contents and compared them with the overall AT content of the whole genomes (consensus of pairwise aligned genomes). Three groups of indels were distinguished: (1) length variable poly-n loci that consist of a single nucleotide that is repeated at least sevenfold, (2) simple sequence repeats (SSRs) that show one repetition of a motif of multiple nucleotides, inverted repeats, or inversions, and (3) indels that do not fall in the former categories.

Further, AT contents of nucleotides adjacent to SNPs were calculated in intervals of increasing size (1–10, 20, 50, and 100 bp in each direction). A script was written in R v.3.0.2, which distinguishes the indels and regions around SNPs, calculates the AT contents, and displays their distributions.

The lineage-specific occurrence of substitutions and microstructural mutations was examined in more detail on the example of group II introns (*atpF*, *rpl16*) that strongly deviated in variability among our four genome pairs. These introns possess a mosaic-like structure of conserved and variable sequence elements. The variable parts usually correspond to the structurally and functionally least constrained terminal stem-loops, which appear in the respective RNA secondary structure. We first annotated the domains of the *atpF* and *rpl16* introns by comparing our sequences with the consensus alignment of Michel et al. [37].The RNA secondary structures of individual domains were then predicted using RNAstructure 5.6 (available at http://rna.urmc.rochester.edu/RNAstructure.html) using the algorithm of Mathews et al. [38]. The “fold as RNA” option was implemented to allow for U–G pairings.

#### Selecting genomic regions as markers for evolutionary studies in *Pyrus*

Our aim was not only to find the most variable plastid regions in *Pyrus* but also to select several regions to be best used in evolutionary studies of *Pyrus*. Thus, efficient amplification and sequencing strategies including primer binding sites, region size and the information content per primer read had to be considered in addition to a high rank in terms of variability. Furthermore, polyA/T stretches larger than seven nucleotides (microsatellites) had to be considered. Their presence usually require two primer reads for sequencing that start from both ends of the amplicon because slippage is likely to occur after the polyA/T stretch. Since a region >1000 bp usually requires two primers to sequence, one microsatellite was not considered a problem, while several microsatellites within the same region led to dismiss it. Considering that current technology generates reliable read lengths of 800–1000 bases, we selected fragments of 900–1300 bp in size ― a size range that can be easily amplified and then sequenced with a maximum of two primers.

## Results and Discussion

### Size and structure of the *Pyrus* plastid genome

**Figure 1:**
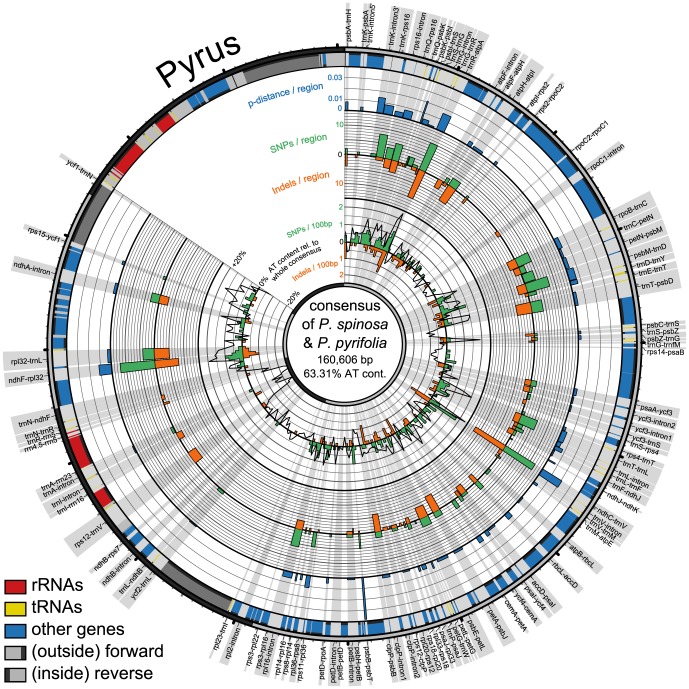
Circular representation of plastid genome pair in *Pyrus*. Shown are consensus sequences of compared species pairs of *Pyrus spinosa* and *P. pyrifolia* with their differing *p*-distances, numbers of SNPs and indels across the consensus. Radial grey highlights show the regions in focus of study with their names. Circular graphs from outside to inside: outermost circle with ticks for every 1,000 bp (small) and 10,000 bp (big) indicates part of genome, single copy regions in light grey and inverted repeats in dark grey; bands show locations of genes (blue), tRNAs (yellow) and rRNAs (red); the three outermost histograms display *p*-distances (blue), number of SNPs (green) and indels (orange) per spacer region; innermost graph shows number of SNPs (green histogram), indels (orange histogram), and AT content relative to the whole consensus (black line graph) of 500 bp long parts of the whole consensus.

**Figure 2:**
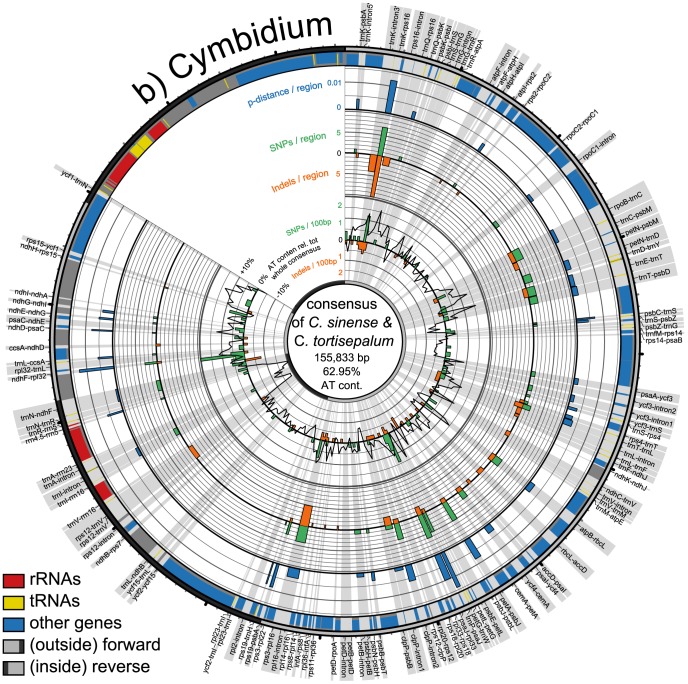
Circular representation of plastid genome pair in *Cymbidium*. Shown are consensus sequences of compared species pairs of *Cymbidium tortisepalum* and *C. sinense* with their differing *p*-distances, numbers of SNPs and indels across the consensus. Radial grey highlights show the regions in focus of study with their names. Circular graphs from outside to inside: outermost circle with ticks for every 1,000 bp (small) and 10,000 bp (big) indicates part of genome, single copy regions in light grey and inverted repeats in dark grey; bands show locations of genes (blue), tRNAs (yellow) and rRNAs (red); the three outermost histograms display *p*-distances (blue), number of SNPs (green) and indels (orange) per spacer region; innermost graph shows number of SNPs (green histogram), indels (orange histogram), and AT content relative to the whole consensus (black line graph) of 500 bp long parts of the whole consensus.

The plastid genome of *Pyrus spinosa* is 159,694 bp in length, and the inverted repeats (IRs) account for 26,396 bp. The large single-copy region (LSC) is 87,694 bp in length and the small single-copy region (SSC) 19,205 bp. The genome has a GC content of 36.6%. Gene content and order are identical to *Pyrus pyrifolia*, with 113 unique genes and 17 duplicates in the IR [30]. The extension of IRs is identical to *P. pyrifolia*, while a 137 bp gap in the LSC of *P. spinosa* directly adjacent to IRa leads to a different IR boundary. The *p*-distance between the two genomes is 0.00145 (Table 2). The consensus structure of the two *Pyrus* genomes and the variability between them is illustrated in Fig. 1. Most of the variation occurs in the noncoding parts, especially in intergenic spacers of the LSC region. The SSC is less variable and almost no variation is found in the IRs. There are some genome parts with intergenic spacers alternating tRNA genes where variation appears to accumulate. This is especially the case in the region from *trnK* to *trnA* and from *rpoB* to *psbD* (Figs. 1, 2).

**Table 2:** Sequence statistics for the four genome pairs compared.

Genome pair	p-distance	Aligned length [bp]	Length difference	SNPs	Indels
*Pyrus spinosa/P. pyrifolia*	0.00145	160607 bp	227 bp	230	173
*Olea europaea/O. woodiana*	0.00294	156091 bp	30 bp	458	112
*Oenothera parviflora/O. argillicola*	0.00122	165952 bp	1690 bp	199	173
*Cymbidium tortisepalum/C. sinense*	0.0008	155833 bp	79 bp	124	62

#### Finding the most variable regions of the *Pyrus* plastid genome

**Figure 3:**
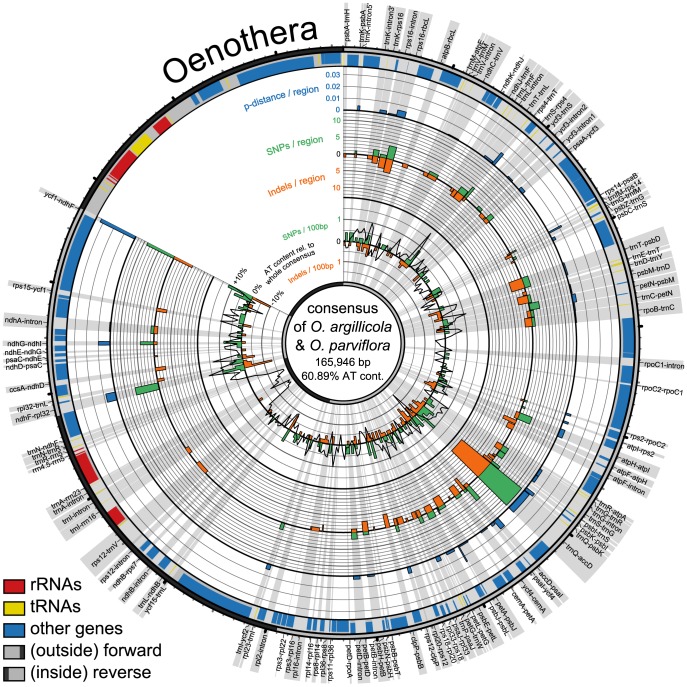
Circular representation of plastid genome pairs in *Oenothera*. Shown are consensus sequences of compared species pairs of *Oenonthera parviflora* and *O. argillicola* with their differing *p*-distances, numbers of SNPs and indels across the consensus. Radial grey highlights show the regions in focus of study with their names. Circular graphs from outside to inside: outermost circle with ticks for every 1,000 bp (small) and 10,000 bp (big) indicates part of genome, single copy regions in light grey and inverted repeats in dark grey; bands show locations of genes (blue), tRNAs (yellow) and rRNAs (red); the three outermost histograms display *p*-distances (blue), number of SNPs (green) and indels (orange) per spacer region; innermost graph shows number of SNPs (green histogram), indels (orange histogram), and AT content relative to the whole consensus (black line graph) of 500 bp long parts of the whole consensus.

**Figure 4:**
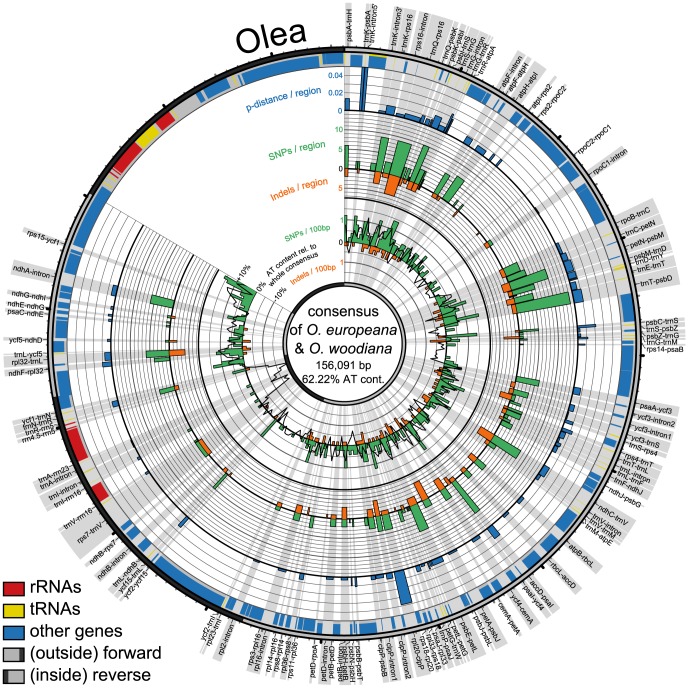
Circular representation of plastid genome pairs in *Olea*. Shown are consensus sequences of compared species pairs of *Olea europaea* and *O. woodiana* with their differing *p*-distances, numbers of SNPs and indels across the consensus. Radial grey highlights show the regions in focus of study with their names. Circular graphs from outside to inside: outermost circle with ticks for every 1,000 bp (small) and 10,000 bp (big) indicates part of genome, single copy regions in light grey and inverted repeats in dark grey; bands show locations of genes (blue), tRNAs (yellow) and rRNAs (red); the three outermost histograms display *p*-distances (blue), number of SNPs (green) and indels (orange) per spacer region; innermost graph shows number of SNPs (green histogram), indels (orange histogram), and AT content relative to the whole consensus (black line graph) of 500 bp long parts of the whole consensus.

The five regions with the highest *p*-distances are the intergenic spacers *psbB–psbT*, *psbI–trnS*, *ndhC–trnV*, *trnR–atpA*, and *ndhF–rpl32*. Taking the PICs as a basis, the five top-ranked regions are *ndhC–trnV*, *trnR–atpA*, *ndhF–rpl32*, *psbM–trnD*, and *trnQ–rps16* (Table 3, Fig. 1–4).

**Table 3:** Ranking and comparison of *p*-distances and differences in the four plastid genome pairs.

	*Pyrus*				*Cymbidium*				*Oenothera*				*Olea*			
Rank	Region	Aligned length [bp]	PICs (SNPs/Indels)	p-distance [*10^−3^]	Region	Aligned length [bp]	PICs (SNPs/Indels)	p-distance [*10^−3^]	Region	Aligned length [bp]	PICs (SNPs/Indels)	p-distance [*10^−3^]	Region	Aligned length [bp]	PICs (SNPs/Indels)	p-distance [*10^−3^]
1	*psbB–psbT*	184	6 (5/1)	37.88	*trnP–psaJ*	366	6 (5/1)	14.04	*ycf1–ndhF*	381	15 (9/6)	36.73	*trnG–trnR*	170	5 (4/1)	23.81
2	*psbI–trnS*	149	4 (3/1)	22.06	*ndhF–rpl32*	259	4 (3/1)	13.04	*psbJ–psbL*	134	2 (2/0)	14.93	*psbC–trnS*	243	6 (5/1)	21.1
3	*ndhC–trnV*	760	24 (12/12)	20.34	*trnK–rps16*	613	17 (7/10)	12.15	*rps4–trnT*	332	5 (4/1)	12.16	*trnR–atpA*	112	3 (2/1)	18.02
4	*trnR–atpA*	909	20 (10/10)	13.61	*psaJ–rpl33*	629	8 (6/2)	9.93	*trnG–trnfM*	172	3 (2/1)	11.9	*trnS–trnG*	715	12 (10/2)	14.33
5	*ndhF–rpl32*	1078	20 (12/8)	11.41	*rps19–psbA*	345	4 (3/1)	8.7	*ndhG–ndhI*	408	5 (4/1)	9.9	*accD–psaI*	702	11 (10/1)	14.27
6	*rpl36–rps8*	459	5 (5/0)	10.89	*rps19–trnH*	122	1 (1/0)	8.2	*accD–psaI*	577	6 (5/1)	8.68	*ycf15–trnL*	354	5 (5/0)	14.12
7	*trnK–rps16*	974	9 (8/1)	8.38	*petA–psbJ*	635	6 (5/1)	7.9	*trnQ–psbK*	355	6 (3/3)	8.52	*psbA–trnH*	447	8 (6/2)	13.51
8	*trnQ–rps16*	905	10 (6/4)	8.3	*ndhD–psaC*	129	1 (1/0)	7.75	*ndhF–rpl32*	932	7 (7/0)	8.26	*rps4–trnT*	326	5 (4/1)	12.31
9	*psbA–trnH*	268	6 (2/4)	7.81	*psbC–trnS*	146	1 (1/0)	6.85	*trnQ–accD*	2615	23 (12/11)	5.59	*trnG–trnM*	174	2 (2/0)	11.49
10	*trnL–trnF*	403	4 (3/1)	7.59	*rrn4.5–rrn5*	168	1 (1/0)	5.95	*rps12–clpP*	397	4 (2/2)	5.13	*psbB–psbT*	186	2 (2/0)	10.75
11	*ndhJ–ndhK*	137	1 (1/0)	7.3	*clpP intron 2*	676	5 (4/1)	5.93	*rps16–rbcL*	976	8 (4/4)	5.06	*trnT–psbD*	1322	15 (14/1)	10.6
12	*rpl14–rpl16*	145	2 (1/1)	6.99	*trnL–ccsA*	180	1 (1/0)	5.56	*atpI–rps2*	216	1 (1/0)	4.63	*trnK–rps16*	899	12 (9/3)	10.06
13	*trnD–trnY*	448	4 (3/1)	6.79	*ndhE–ndhG*	185	1 (1/0)	5.41	*rps2–rpoC2*	219	2 (1/1)	4.59	*rps2–rpoC2*	209	4 (2/2)	9.66
14	*psbM–trnD*	1235	11 (8/3)	6.51	*trnS–psbZ*	230	1 (1/0)	4.35	*trnP–psaJ*	515	4 (2/2)	4.37	*trnS–rps4*	314	3 (3/0)	9.55
15	*trnW–trnP*	156	1 (1/0)	6.41	*ndhG–ndhI*	233	1 (1/0)	4.29	*trnI–ycf2*	462	2 (2/0)	4.33	*psbN–psbH*	105	1 (1/0)	9.52
16	*rpl16 intron*	1003	9 (6/3)	6.01	*trnK–psbA*	257	1 (1/0)	3.89	*atpH–atpI*	939	5 (4/1)	4.26	*trnD–trnY*	107	2 (1/1)	9.43
17	*ycf4–cemA*	526	3 (3/0)	5.7	*trnS–rps4*	287	1 (1/0)	3.48	*trnK intron 5′*	249	2 (1/1)	4.03	*psbM–trnD*	657	7 (6/1)	9.15
18	*rbcL–accD*	569	6 (3/3)	5.33	*rpl16 intron*	1191	9 (4/5)	3.46	*petN–psbM*	926	3 (3/0)	3.24	*trnQ–psbK*	335	5 (3/2)	9.01
19	*trnT–trnL*	1241	8 (6/2)	4.94	*trnT–trnL*	610	4 (2/2)	3.36	*psaA–ycf3*	669	3 (2/1)	3.01	*atpI–rps2*	222	2 (2/0)	9.01
20	*psaI–ycf4*	413	4 (2/2)	4.89	*atpI–rps2*	300	1 (1/0)	3.33	*trnS–psbZ*	348	1 (1/0)	2.87	*atpF intron*	697	7 (6/1)	8.62
21	*rps8–rpl14*	207	2 (1/1)	4.83	*psbB–psbT*	323	1 (1/0)	3.1	*trnK–rps16*	758	4 (2/2)	2.67	*petN–psbM*	1171	12 (10/2)	8.62
22	*rpl33–rps18*	218	2 (1/1)	4.67	*trnQ–psbK*	348	1 (1/0)	2.87	*petD intron*	761	3 (2/1)	2.65	*trnK–psbA*	236	2 (2/0)	8.47
23	*trnS–trnG*	651	3 (3/0)	4.62	*ycf4–cemA*	728	3 (2/1)	2.75	*trnS–trnG*	788	2 (2/0)	2.54	*ndhF–rpl32*	479	4 (4/0)	8.37
24	*rps16 intron*	909	7 (4/3)	4.48	*rps4–trnT*	367	2 (1/1)	2.74	*trnG intron*	804	4 (2/2)	2.5	*psbI–trnS*	120	1 (1/0)	8.33
25	*petD–rpoA*	225	1 (1/0)	4.48	*trnT–psbD*	947	2 (2/0)	2.11	*psaI–ycf4*	412	5 (1/4)	2.45	*petG–trnW*	121	1 (1/0)	8.26
26	*atpF–atpH*	451	3 (2/1)	4.44	*atpB–rbcL*	960	3 (2/1)	2.11	*psbE–petL*	984	4 (2/2)	2.05	*rps14–psaB*	122	1 (1/0)	8.2
27	*trnM–atpE*	242	2 (1/1)	4.29	*petN–trnD*	1020	2 (2/0)	1.96	*trnT–trnL*	1114	4 (2/2)	1.89	*psaA–ycf3*	741	6 (6/0)	8.1
28	*psaJ–rpl33*	472	4 (2/2)	4.29	*trnE–trnT*	1216	3 (2/1)	1.68	*trnT–psbD*	1441	6 (2/4)	1.43	*trnK intron 5′*	268	3 (2/1)	7.49
29	*rpoB–trnC*	1216	8 (5/3)	4.13	*psaA–ycf3*	638	1 (1/0)	1.57	*ycf3 intron 2*	720	1 (1/0)	1.39	*rpl32–trnL*	835	10 (6/4)	7.26
30	*trnL intron*	514	3 (2/1)	3.9	*rpoB–trnC*	1461	3 (2/1)	1.38	*petB intron*	775	4 (1/3)	1.34	*ndhC–trnV*	1119	12 (8/4)	7.24

The regions are sorted according to *p*-distances.

Comparing our results with the ranking of Shaw et al. [9] it appears that 17 of our 30 top-ranked regions in *Pyrus* are also among the 32 top-ranked in their study. However, their ranks are different. For example, in Shaw et al. [8], the *rpl32–trnL* spacer has the highest number of PICs whereas it is only at rank 8 in *Pyrus*. The *trnR–atpA* spacer, which has the second-highest number of PICs in *Pyrus*, was not at all reported. However, the ranking of Shaw et al. may not be that comparable because the authors “normalized” their PICs with the aim to reduce the influence of different evolutionary rates or genetic distances. They divided the number of PICs within a region from a certain taxonomic lineage by the total sum of PICs within the same lineage. Therefore, their results do not directly show lineage-specific differences in marker variability, although the absolute variability of a given genomic region is the only relevant fact in any analysis.

Low genetic distances in *Pyrus* have been pointed out in two earlier studies of *Pyrus* plastid genomes [27], [39]. These studies were motivated by the horticultural importance of *Pyrus*, and focused on Asian species and cultivars. Katayama and Uematsu [39] provided a physical map of the plastid genome of *Pyrus ussuriensis* var. *hondoensis* and ran an RFLP analysis on cpDNAs from 11 accessions of five *Pyrus* and two *Prunus* species. However, there were no sequence data to support their conclusions. Terakami et al. [27] aligned the three plastid genomes of *Pyrus pyrifolia*, *Malus* × *domestica*, and *Prunus persica*. The authors calculated the proportion of mutational events using the same formula as Shaw et al. [8] for 89 noncoding regions, and ranked the compared regions according to their variability comparing *Pyrus* with *Malus* and *Prunus* (ingroup and outgroup were not specifically defined). While the *ndhC–trnV* and *trnR–atpA* spacers depict the highest sequence divergence in both, Terakami et al. and our work presented here, the overall rankings are strongly different. Terakami et al. found the spacers *rpl33–rps18*, *psbI–trnS*, and *rpl14–rpl16* from the third to fifth rank. In our *Pyrus* ranking, these spacers are at positions 22, 2, and 12 (based on *p*-distances) and 43, 22, and 41 (based on PICs), respectively. These differences may be explained by the much greater distance between the *Pyrus* and *Malus* plastid genomes than our two *Pyrus* genomes. The crown group of *Pyrus* diversified 27–33 mya while the crown group of *Malus* was inferred to have diversified 34–46 mya [40].

Various plastid regions have also been sequenced for a large number of samples in *Pyrus.* Katayama et al. [41] sequenced the *rps16–trnQ* and *accD–psaI* spacers and reconstructed a network based on 25 different haplotypes including 21 species of *Pyrus* and multiple individuals of *P. pyrifolia* and *P. ussurienis*, respectively. The authors found both spacers to contain highly variable AT-rich mutational hotspots and concluded that these regions are “hypervariable”, while their remaining *Pyrus* sequences showed hardly any variation. The authors argued that their results confirmed their earlier hypothesis of strong sequence conservation in the plastid genomes of *Pyrus* [39]. No explanation, however, was given why particularly the *rps16–trnQ* and *accD–psaI* spacers had been chosen and not one of the highest ranked ones in terms of variability. The authors noted that the frequency of microstructural mutations in both spacers studied was markedly higher than of substitutions and that haplotypes were mostly defined by indels. Such a dominance of microstructural mutations over substitutions is typical of AT-rich sequence elements that constitute terminal stem-loops of introns and transcribed spacers which are often unique to small lineages of plants [11]. At the same time such sequence elements often exhibit high levels of homoplasy. Thus, the exclusive application of these elements to calculate networks or trees may potentially lead to wrong conclusions. Wuyun et al. [42] sequenced the *rps16–trnQ* and *accD–psaI* spacers to reconstruct a phylogenetic network of *Pyrus ussuriensis* in China, which was largely based on the presence or absence of indels in the two spacers. Compared with our results, the two regions used by Katayama et al. [47] and Wuyun et al. [48] are also not the most variable plastid regions in *Pyrus*: the *trnQ–rps16* spacer ranks at place 24 for *p*-distances and at place 5 for PICs. The *accD–psaI* spacer ranks at place 18 for *p*-distances and at place 20 for PICs.

### Plastid markers proposed for *Pyrus*

Four intergenic spacers of 900 to 1000 bp and the *rpl16* group II intron (ca. 1000 bp) are proposed here to be sequenced for evolutionary studies in *Pyrus* (Table 4). They were selected from the most variable genomic regions (Table 3) considering an efficient sequencing strategy (see methods section).

**Table 4:** Genomic regions proposed for evolutionary analyses in *Pyrus* and primers for their amplification.

Region	Amplified fragment	Primer name	Primer sequence	Reference
*ndhC–trnV*	900 bp	ndhC–F	TGCCAAAATAGGAATAACAC	Goodson et al. [46]
		PYRtrnV–150R	CCACATAATGAATCAGAGCAC	this study
*trnR–atpA*	1000 bp	trnR–F	GTCTAATGGATAGGACAGAGG	this study
		atpA–180R	GGAACRAACGGYTATCTTGATTC	this study
*psbM–trnD*	1350 bp	PYRpsbM–F	CCTTGGCTGACTGTTTTTACG	this study
		PYRtrnD–R	GAGCACCGCCCTGTCAAGG	this study
*trnQ–rps16*	900 bp	trnQ (UUG)	GCGTGGCCAAGTGGTAAGGC	Shaw et al. [9]
		rps16x1	GTTGCTTTCTACCACATCGTTT	Shaw et al. [9]
*rpl16* intron	1300 bp	PYR–rps3F	GATTATTGTTCCTATGCAG	this study
		PYR–rpl16R	GCTTGAAGAGCATATCTAC	this study

**Figure 5:**
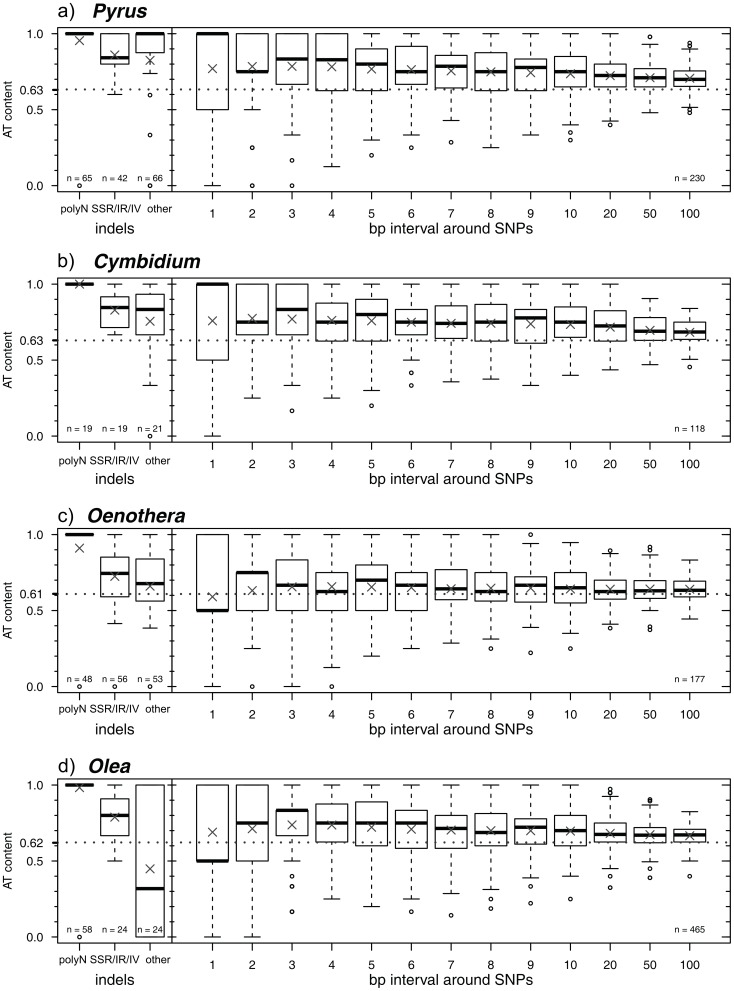
Mutational dynamics in group II introns. a) Schematic consensus structure of plastid group II introns based on Michel et al. (1989). Roman numbers indicate the six domains. B) Alignment and predicted RNA secondary structure for domain IV of the *atpF* intron in *Cymbidium*, *Pyrus*, *Oenothera* and *Olea*. The apparently non-homologous sequence blocks are placed separately in the alignment. There are no substitutions or length mutations in *Pyrus* and *Cymbidium*, the structures shown are therefore identical in the two species compared. The shown secondary structures of *Oenothera* and *Olea* are consensus structures. Two conserved nucleotide blocks at the 3′ and 5′ ends, indicated by thick blue bars, are conserved across all taxa and homologous in primary sequence and secondary structure. These conserved sequence blocks form the stem of the domain while variation occurs in the terminal stem-loops part of the domain. c) Alignment and predicted secondary RNA structures of domain IV of the *rpl16* intron. For clarity, only the part of the domain with positions variable within genera are shown; “[-]” mark the omitted stem-loop elements. The apparently non-homologous sequence blocks are placed separately in the alignment. Those positions where variation occurs within a genus are marked with arrows. See text for more explanation.

Among the regions with a minimum size of 500 bp, the *ndhC–trnV* and *trnR–atpA* spacers rank 3^rd^ and 4^th^ according to *p*-distances, and *ndhC–trnV* has the highest number of PICs. Both can be sequenced with just one primer (either forward or reverse). Thus, these spacers are especially useful if large sample numbers need to be analysed. The *ndhF–rpl32* spacer (ranked 3^rd^ of the regions >500 bp in Table 4) was not considered further because there are two large microsatellites. This fragment can therefore not be sequenced with two primers. The same problem occurs in the *rps16–trnK* spacer (ranked 4^th^ of the regions >500 bp in Table 4) where two poly G and one poly T are likely to cause sequencing problems with pherograms unreadable after the homonulceotide stretches. The *trnQ*-*rps16* and *psbM*-*trnD* spacers follow in the ranking. Both also have polyA/T microsatellites. While they can be covered with two primer reads that overlap at the microsatellite, they may not be as efficiently sequenced than the *ndhC–trnV* and *trnR–atpA* spacers for large sample numbers. The *rpl16* intron (ranked at 7^th^ position of the regions >500 bp in Table 4), is particularly recommended because it was shown to also possess a high phylogenetic structure *R* in different angiosperm sequence data sets [43]–[45]. Multiple *rpl16* sequence alignments can therefore be expected to yield well-resolved and well-supported trees also in *Pyrus*. The intron can be co-amplified with the *rpl14–rpl16* spacer. The use of the reverse primer PYR-rpl16R (Table 4) will allow to sequence the whole intron with one read. The *rpl16* intron contains a polyA/T stretch of variable length in different species of *Pyrus* (see also Fig. 5c), what implies that an additional forward primer read may be necessary to cover the whole intron in some samples.

Primers were newly designed for *trnR*-*atpA* as this region to our knowledge has never been used in any evolutionary study so far. For *ndhC*-*trnV*, primers were available [46] but we designed a new *Pyrus*-specific reverse primer in order to completely cover the spacer-exon boundary. For *trnQ*-*rps16*, the universal primers designed by Shaw & al. [9] work for *Pyrus* as well. Available primers for *psbM*-*trnD* [47] were re-designed for *Pyrus* to avoid mismatches in the forward and then to obtain a similar melting temperature in the reverse primer. For the *rpl16* intron, primers were also adapted to *Pyrus* following the general amplification strategy of [43] and [44] with a forward primer that anneals to the *rps3* exon. This ensures that the *rpl16* intron can be amplified and sequenced completely. The universal reverse primer rpl16R [48] was replaced by a *Pyrus*-specific primer that anneals further downstream to cover the intron-exon boundary.

#### Comparison of plastid genomes with low *p*-distances in angiosperms

In addition to *Pyrus*, we explored variability patterns in plastid genome pairs of *Oenothera argillicola* and *O. parviflora* (Onagraceae), *Olea europaea* and *O. woodiana* (Oleaceae), and *Cymbidium sinense* and *C. tortisepalum* (Orchidaceae) which have comparable low *p*-distances (Table 2). The variability patterns of all four genome pairs are illustrated using a Circos-plot (Figs. 2–4). Each genome pair has different regions with highest *p*-distances and highest numbers of PICs, resulting in a genome pair-specific ranking (Table 3). The results of the pairwise comparisons of individual introns and spacers for each genome pair are provided in Table S2.

The SNPs and indels are almost evenly spread across the LSC and the SSCs in *Olea*. In *Cymbidium*, SNPs and indels are more clustered. The plastid genomes of *Pyrus* and *Oenothera* exhibit strong variation in certain areas, e.g. between *trnT* and *rpoB* (Figs. 1, 3) but alsoalso homogeneously distributed mutations across their genomes. The *Olea* genome stands out by many more SNPs than indels, while the other genomes have almost as many indels as SNPs.

In our summary of the 30 most variable genomic regions including all four genome pairs,77 different regions appear in total (Table 3). It is noteworthy that only two spacers, *ndhF–rpl32* and *trnK–rps16*, are consistently placed among the 30 most variable regions. Eight spacers appear three times: *atpI–rps2*, *psaA–ycf3*, *psbB–psbT*, *rps4–trnT*, *trnQ–psbK*, *trnS–trnG*, *trnT–psbD*, and *trnT–trnL*.

#### Earlier comparisons of plastid genomes in angiosperms for marker selection

In an approach to explore hitherto unused plastid regions as phylogenetic markers, Shaw et al. [9] in 2007 compared whole plastid genomes in a comprehensive way. They analysed genome pairs from three different lineages of angiosperms [*Atropa* and *Nicotiana* (Solanaceae) for the asterids, *Lotus* and *Medicago* (Fabaceae) for the rosids, and *Oryza* and *Saccharum* (Poaceae) for the monocots]. They found nine previously unexplored plastid regions with high levels of variation based on the numbers of PICs: *rpl32–trnL*, *trnQ–rps16*, *ndhC–trnV*, *ndhF–rpl32*, *psbD–trnT*, *psbJ–petA*, *rps16–trnK*, *atpI–atpH*, and *petL–psbE*. As noted before, we were interested to compare the distance levels of these genomes to the genome pairs examined here, as we expected considerable differences. The *p*-distances were indeed much higher and are here calculated as follows: *Lotus japonicus*/*Medicago truncatula p* = 0.17603, *Nicotiana tabacum*/*Atropa belladonna p* = 0.01363, *Saccharum* hybrid/*Oryza sativa p* = 0.04879.

Another comparative study of plastid genomes was carried out by Dong et al. [13] five years later.They looked at 14 angiosperm genera for which more than one plastid genome was available, again with the goal of finding markers for phylogeny reconstruction and DNA barcoding. They concluded that *ycf1*, *psbA–trnH*, *rpl32–trnL*, *trnQ–rps16*, *ndhC–trnV*, *trnK*/*matK*, and *trnS–trnG* are best-suited.

Next generation sequencing has resulted in an increased availability of plastid genome data in recent years (Table 5) that were used to find markers for various phylogenetic analyses in certain angiosperm lineages, to recover promising regions for haplotype studies or to differentiate closely related species and cultivars [14], [21], [22], [24]–[27], [49]–[52]. None of the authors addressed more general patterns of plastid genome mutational dynamics and molecular evolution. As noted before, the studies span an enormous range of different genetic distances in the genomes compared. The compared economically important asterids (e.g., *Solanum*, *Nicotiana*, *Lactuca*) are well represented while studies on other taxa are still scarce. Moreover, the approaches and methods applied in these studies differ. Most of them calculated some kind of sequence variability, while others additionally or solely reconstructed phylogenetic trees based on small taxon sets to assess the phylogenetic utility of these regions. A spectrum of 37 plastid loci was reported as “highly variable” in the studies cited above. Most commonly mentioned were *rpl32–trnL* (7x), *trnQ–rps16* (5x) *trnK–rps16* (4x), and *ndhC–trnV* (4x). Nevertheless, the question remains how representative the earlier pairwise genome comparisons are, and to what extent their conclusions are also valid for other families and genera of flowering plants.

**Table 5:** Identification of most variable plastid regions based on pairwise genome comparisons across angiosperms.

Reference	Taxa studied	Markers found as most variable
Daniell et al. [52]	Asterids: *Atropa belladonna*, *Nicotiana tabacum*, *Solanum bulbocastanum*, *S. lycopersicum* (Solanaceae)	*psbK–psbI, rps12–clpP, trnG–trnfM, trnK–rps16, trnQ–rps16*
Timme et al. [49]	Asterids: *Helianthus annuus*, *Lactuca sativa* (Asteraceae)	*ndhC–trnV, rpl32–trnL, rps12–clpP, trnE–rpoB, trnY–trnE*
Shaw et al. [9]	Angiosperms: Asterids: *Atropa belladonna*, *Nicotiana tabacum* (Solanaceae), Rosids: *Lotus*, *Medicago* (Fabaceae), Monocots: *Oryza*, *Saccharum* (Poaceae)	*rpl32–trnL trnQ–rps16 ndhC–trnV, ndhF–rpl32, psbD–trnT, psbJ–petA, rps16–trnK, atpI–atpH, petL–psbE*
Doorduin et al. [50]	Asterids: *Jacobaea vulgaris*, *Helianthus anuus*, *Lactuca sativa*, *Parthenium argentatum*, *Guizotia abyssinica* (Asteraceae)	*ndhC–trnV, ndhC–atpE, rps18–rpl20, clpP, psbM–trnD*
Gargano et al. [51]	Asterids: *Solanum tuberosum* subsp. *tuberosum*, S. *bulbocastanum* (Solanaceae)	*ndhA intron, petN–psbM, rpl32–trnL, rps2–rpoC2, trnQ–rps16*
Yang et al. [24]	Monocots: *Cymbidium* (Orchidaceae)	*cemA–petA, clpP–psbB, ndhF–rpl32, petA–psbJ, psbA–trnK, rpl32–trnL, trnE–trnT, trnK–rps16, trnL–ccsA, trnP–psaJ, trnT–trnL*
Dong et al. [13]	Angiosperms: *Acorus* (Acoraceae), *Aethionema* (Brassicaceae), *Calycanthus* (Calycanthaceae), *Chimonanthus* (Calycanthaceae), *Eucalyptus* (Myrtaceae), *Gossypium* (Malvaceae), *Nicotiana* (Solanaceae), *Oenothera* (Onagraceae), *Oryza* (Poaceae), *Paeonia* (Paeoniaceae), *Populus* (Salicaceae), *Solanum* (Solanaceae)	*ycf1, trnH–psbA, rpl32–trnL, trnQ–rps16, ndhC–trnV, trnK/matK, trnS–trnG*
Ku et al. [26]	Asterids: *Catharanthus roseus* (Apocynaceae), *Asclepias syriaca* (Apocynaceae), *Coffea arabica* (Rubiaceae), *Solanum lycopersicon* (Solanaceae)	*ndhF–rpl32, rpl32–trnL, rps16–trnQ, trnE–trnT, trnK–rps16*
Ku et al. [25]	Asterids: *Ardisia polysticta* (Primulaceae – Myrsinioideae) *Panax ginseng* (Araliaceae) *Sesamum indicum* (Pedaliaceae)	*ccsA–ndhD, ndhG–ndhI, rpl14–rpl16, rpl32–trnL, trnK–rps16*
Särkinen & George [14]	Asterids: *Solanum tuberosum*, *S. bulbocastanum*, *S. lycopersicum* (Solanaceae)	*atpB–rbcL, clpP–psbB, ndhF, ndhF–rpl32, petL–psaJ, petN–psbM, rpl32–trnL, rpoC1–rpoB, trnA–trnI, trnK–rps16, ycf1*

Shaw et al. [8] assumed a high universality of their results. But Daniell et al. [52], who compared plastid genomes of Solanaceae, found spacers with higher sequence divergence not mentioned in [8]. Timme et al. [49] analysed Asteraceae and indicated that their ranking of most variable regions barely overlapped with the ranking of Shaw et al., and suspected that “each family or major lineage will most likely have a unique set of variable regions” [43]. Shaw et al. [9] in 2007 found no less than 11 new highly variable markers not considered in their 2005 study therefore pointed to the need of a test-wise screening of the “universal” regions to find the most suitable one for a given lineage. Likewise, Dong et al. [13] stated that markers useful for one group may not be useful for another and recommended evaluating markers in detail before selecting them for further use. With the aim of resolving the species tree in the huge genus *Solanum*, Särkinen and George [14] found that the average amount of variable characters differs within subclades of the genus. In their view, the degree to which the utility of a marker can be extended to more inclusive clades would then also be clade-specific.

In summary, lineage specific differences in variability and phylogenetic utility of plastid genomic regions were reported in various cases in flowering plants although there was never any standardized comparative approach to better understand this issue. Moreover, none of the previous studies explicitly addressed phylogenetic signal as being different from similarity-based variability, or looked at any molecular evolutionary characteristics.

#### Molecular evolution and lineage specific variability of genomic regions

**Figure 6:**
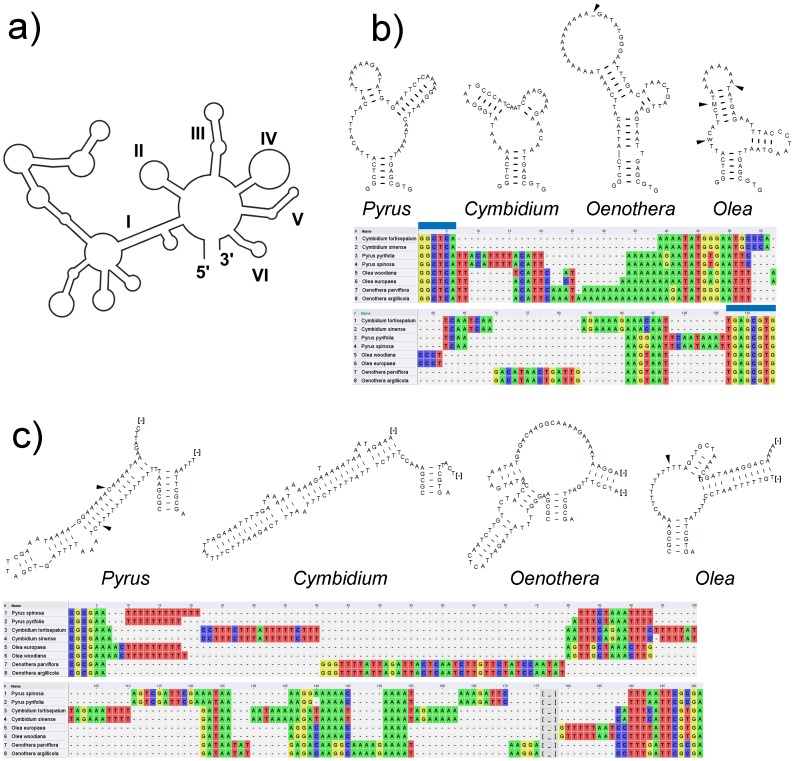
AT content of indels and areas around substitutions. Boxplot representation of the AT content in different types of indels (polyN, short sequence repeats (SSR) and other indels) on the left side and in areas with different sizes around all substitutions (SNPs) in the genome on the right side for a) *Pyrus spinosa* and *P. pyrifolia*), b) *Cymbidium tortisepalum* and *C. sinense*, c) *Oenonthera parviflora* and *O. argillicola* and d) *Olea europaea* and *O. woodiana*. The cross in each boxplot indicates the mean of the distribution, the thick line refers to the median. The dotted line shows the AT content of the whole consensus sequence.

Lineage-specific differences in variability are often explained by patterns of molecular evolution. It has been exemplarily demonstrated for regions such as *psbA–trnH* [53] or *trnL–trnF* [54] that variability is strongly influenced by structural constraints. Empirical analysis of *petD* group II intron sequences has further shown that increased length correlates with increased AT strongly influenced byal constraints Empirical analysis of *petD* group II intron sequences has further shown that increased length correlates with increased AT content [12]. Figure 5 shows the AT contents of three types of indels (left side) and around SNPs (right side) in intervals of increasing size of each of our genome pairs. AT content distributions are displayed in boxplots with the cross showing the mean and the thick line referring to the median. Respective boxplots arranged along the *x*-axis then depict maximum distances of the intervals in each direction of the SNP. Apart from rare exceptions the surroundings of SNPs are distinctly more AT-rich than the whole genome (Fig. 6), indicating that substitutions occur predominantly in AT-rich stretches. The AT contents of the consensus sequences are displayed as dotted lines. Looking at indels, considerable differences are apparent in the frequency of different kinds among the four plant lineages. In *Olea*, length-variable polyA/T stretches are most common. In *Oenothera*, all three kinds of indels occur with almost equal frequency, while in *Cymbidium* and *Pyrus* indels without a clear motif predominate.

The AT content is significantly increased in sequence elements affected by microstructural changes (Fig. 6), both in SSRs and in the non-SSR indels. The SSRs are generally AT-rich, so the templates for these SSRs must be AT-rich as well. And therefore, their frequency is also significantly higher in AT-rich sequence elements. It can thus be suggested that mutational dynamics is increased in AT-rich sequence. A strong correlation between high AT content and high substitutional rates was also recently demonstrated in plastid genomes of Lentibulariaceae [55].

Comparative studies of the molecular evolution of group II introns showed substitutions, length-variable homonucleotide stretches and indels to predominantly occur in domains I, III and IV. These domains are also the most variable with respect to size and experience less strong functional constraints compared to the other domains [12], [56], [57]. Furthermore, considerable variation occurs in sequence elements that are unique to certain lineages, where they have evolved through stepwise insertion processes connected to the formation of stable helical elements [11]. In our data set, this is for example evident in the *petD* and *rpl16* introns. They appear at strikingly different positions in the rankings of the respective genome pairs (Table 3 and S2). In both introns the variation between the sequences of a genome pair is mostly caused by length variable polyA/T stretches or AT-rich indels.

Domain IV of the *atpF* intron belongs to a conserved group II intron (Fig. 5a) with no variation between the *Cymbidium* and *Pyrus* sequences, two substitutions in *Olea* and a length-variable polyA-stretch in *Oenothera* (Fig. 5b). The alignment (Fig. 5b) illustrates two conserved sequence blocks that are homologous and conserved across all genera. They form the stem of the domain. Terminal parts of the domain such as the length-variable polyA-stretch in *Oenothera* have no structural constraints and therefore evolve rather freely. In *Olea*, there are two substitutions (indicated with ambiguity codes in the secondary structure) and one length variable polyA stretch. Again they occur in the terminal stem-loop and have no influence on the structure. The *rpl16* intron is more variable in *Pyrus* than in the other genome pairs. The polyT-stretch of *Olea* and *Pyrus* (beginning at position 10) is hypothesized as homologous in the alignment. But the predicted secondary structures (Fig. 5c) show that this polyT stretch forms different secondary structures caused by the different adjacent sequence elements. In *Olea*, it forms a bulge but in *Pyrus* it forms a stem-element together with a complementary ‘AAAACACAAAAAA’ motif [12], [54].

#### Sequence variability versus phylogenetic signal

It is important to note that sequence variability as such does not necessarily correlate with the amount of hierarchical phylogenetic signal in a multiple sequence matrix. Thus, *p*-distances and PICs―which are both measures of sequence variability and describe the similarity of sequences―will not necessarily indicate the phylogenetically most informative regions. The phylogenetic utility of genomic regions depends on the distribution and kind of character state transformations throughout the evolutionary history of the sequences. Several statistics have been proposed to measure the hierarchical phylogenetic signal (referring to the phylogenetic structure in a data set) that take into account the number of resolved nodes and the statistical support for these nodes [58], [59]. Specifically, the statistics *R*, *B*, and *C*, have been defined by Müller et al. [59]. The most important one, *R*, measures the proportion of resolved clades and their support in a tree inferred from a given data set relative to the maximum possible resolution and support. If all nodes have maximum support, *R* will get the value 1; if the phylogeny is completely unresolved (consists only of polytomies), *R* will have the value 0.

The empirical evaluation of phylogenetic structure in a genomic region generally requires a multiple sequence alignment of a representatively sampled clade. From the datasets that have been evaluated in detail using the *R* statistic [44], [45], [59], it is evident that at one hand higher variability often leads to more phylogenetic information (simply because there are more potentially informative characters). On the other hand, there are marked differences in the quality of hierarchical phylogenetic signal coming from the same number of variable positions in different kinds of genomic regions [45]. These can be explained by different molecular evolutionary patterns. The general trend across angiosperms is that high phylogenetic structure is found in intergenic spacers and group I and II introns, but not in protein-coding genes except *matK*. In our case of very closely related plastid genomes, the effects of multiple changes of the same site, eventually leading to saturation, or reversals, will probably not be very significant because these sequences are just starting to diverge. Nevertheless, it will be interesting to determine the phylogenetic structure in the top-ranked genomic regions in terms of variability once more extensive taxon sets will be available.

Moreover, highly variable regions will be needed to distinguish haplotypes (or species), even if they do not provide sufficient information about their phylogeny [44]. If haplotypes are used in the sense of individual alleles, the pure variability is most important. However, AT-rich sequence elements (often in stem-loops) can be highly homoplastic with respect to the evolution of microstructural mutations [60], [61]. The most extreme causes of homoplasy are inversions [62], [63]. Therefore, especially those markers that contain a single AT-rich mutational hotspot should be tested for congruence in signal with other plastid markers. Haplotype analyses often only use one or two markers, but experiences from other studies that have successfully reconstructed evolutionary relationships among closely related species indicate that the combination of four or five regions will be needed. An increased number of characters increases resolution and support also in network analyses [64], [65].

#### Implications for plastid marker development in angiosperms

About 20–30 plastid spacers and introns are regularly sequenced for phylogenetic and haplotype analyses, for which universal amplification primers exist. Also, considerable progress has been made during recent years in predicting phylogenetic utility from molecular evolutionary patterns, revealing differences in phylogenetic structure of genes, group I and group II introns, and intergenic spacers [10]–[12], [45], [59]. In this way, markers with high versus low phylogenetic signal can be distinguished. For higher levels of genetic distance levels (e.g. distantly related species, genera, and families of flowering plants), a detailed evaluation of markers is therefore hardly necessary because sound predictions can be made. But is it worth to sequence whole plastid genomes when very closely related groups of species are to be studied?

Our comparison of genome pairs at comparable low distances shows that the mutational dynamics of plastid genomic regions may follow its own path in different lineages. While the variability in the respective unique sequence elements contributes the major proportion of the overall variability of a genomic region at that level, this contribution will be increasingly negligible at higher distance levels. The exploration of the plastid genome for the most variable and most suitable regions will therefore be a worthwhile investment when genetic distances are low.

It is of course possible to sequence all or at least most of the 30 promising plastid regions individually for a small taxon set in a given group. However, the effort needed is quite high. At least 60 individual fragments would need to be PCR-amplified and sequenced using many individual primers. Since only three to five loci are usually sequenced in evolutionary studies, a large part of these data would be wasted or deposited in GenBank as “unpublished”. The sequencing and assembly of whole plastid genomes is still laborious, especially if critical areas of low coverage or homonucleotide stretches are verified by Sanger sequencing. Often overlooked costs have to be considered as well: this includes higher requirements for IT hardware and much increased time for sequence assembly and data management compared to traditional sequencing. Still, sequencing a complete plastid genome has many benefits over many single-marker PCRs. First, the complete genome sequence ensures that all genomic regions can be considered for marker development. And second, generating complete genomes allows for using the genome sequence for other studies, so that data are added in a complementary way to build proper information sources for the respective lineages (e.g., for comparative genomics, primer design, detection of plastid microsatellites, or extraction of regions for phylogenetic studies). We therefore conclude that whole plastid genome sequencing will remain a worthwhile approach for marker development in evolutionary studies of plants.

## Supporting Information

Table S1 **Primers for verification of sequence parts ambiguously read by the 454 sequencing.**
(XLSX)

Table S2 **Ranking of all regions for the four genome pairs.**
(XLSX)

File S1 **Pairwise alignment of the plastid genomes of** ***Pyrus spinosa*** **and** ***P. pyrifolia.***
(FASTA)

File S2 **Pairwise alignment of the plastid genomes of** ***Cymbidium tortisepalum*** **and** ***C. sinense.***
(FASTA)

File S3 **Pairwise alignment of the plastid genomes of** ***Oenothera parviflora*** **and** ***O. argillicola.***
(FASTA)

File S4 **Pairwise alignment of the plastid genomes of** ***Olea woodiana*** **and** ***O. europaea.***
(FASTA)
